# Supplementary material for: Position effects of 22q13 rearrangements on candidate genes in Phelan-McDermid syndrome
Source: PLoS One. 2021 Jul 6;16(7):e0253859. doi: 10.1371/journal.pone.0253859 (PMC8259982; doi:10.1371/journal.pone.0253859)
Supplement: S2 File — Notes for sheets 1–4 in the S1 File. Fig 1. Deletion breakpoints for 91 individuals with PMS used for TAD analysis. Fig 2. Parametric analysis of Biolog plates 1–8 for individuals PMS 1 through PMS 5 versus 50 controls. Table. Genes selected for this study with their aliases and functions. Information from the Gene database from NCBI website. (DOCX) [file pone.0253859.s002.docx]

Notes for sheet 1 through sheet 4 in the S1 file

Only data where an array is available are provided. Selected parent-provided health data, physician-provided physical exam, and physician-recorded growth percentiles are included. The columns describe the phenotype. Unless otherwise specified, “yes” and “no” responses are whether the patient currently has the condition, although that was not always clear from how the questionnaires were recorded. Height and Head Circumference (HC) are provided as age and gender adjusted percentiles.

**Fig 1.** Deletion breakpoints for 90 individuals with PMS used for TAD analysis


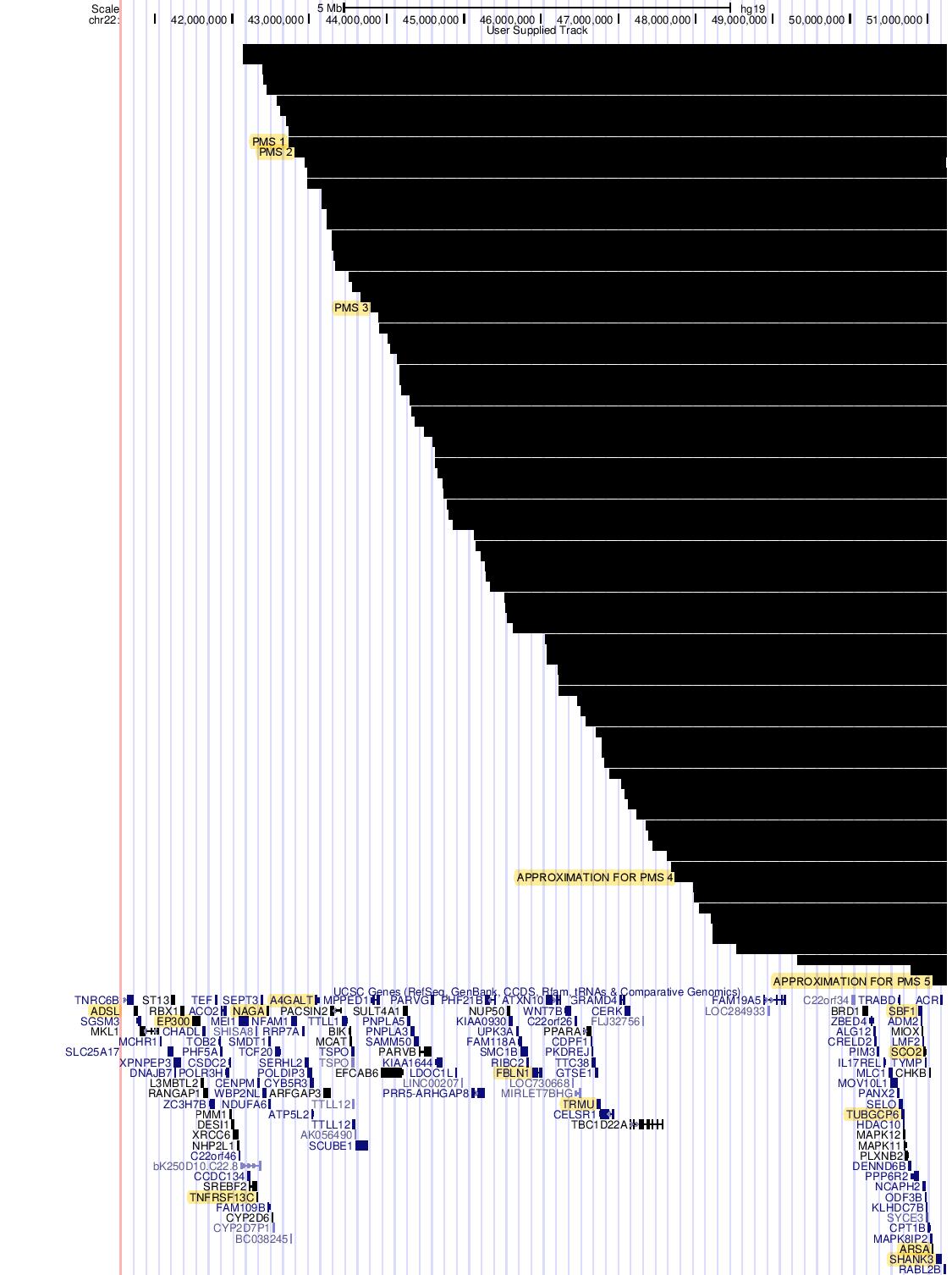


**Fig 2.** Parametric analysis of Biolog plate PM-M1 for patients 1 through 5 versus 50 controls

PMS 1


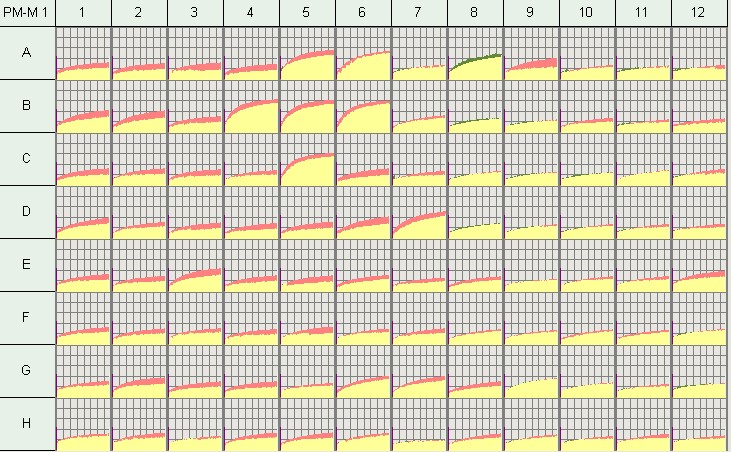


PMS 2


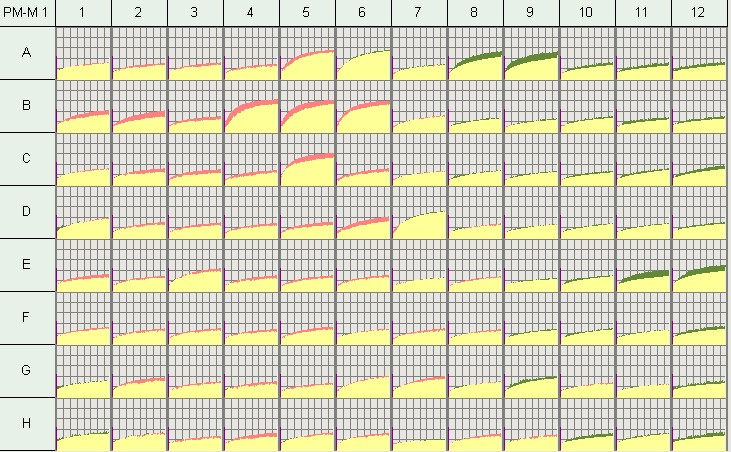


PMS 3


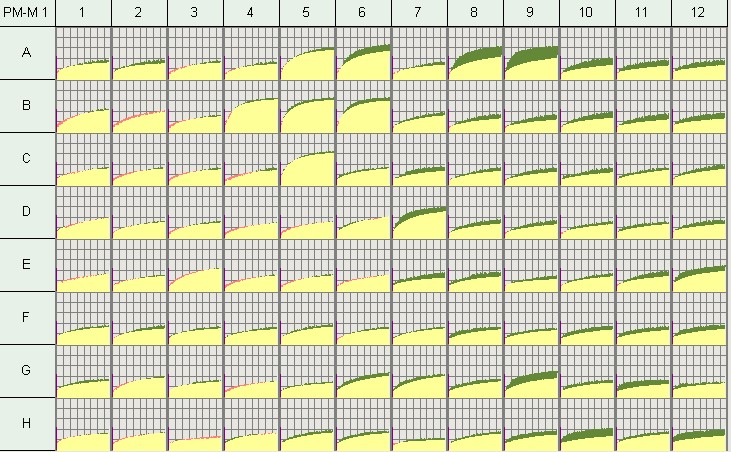


PMS 4


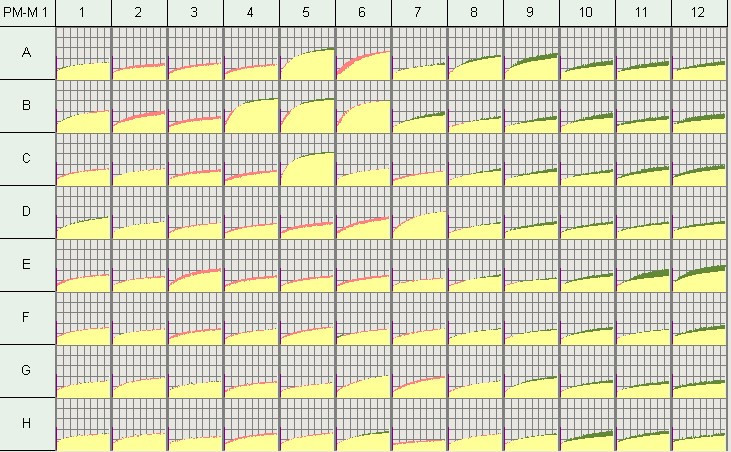


PMS 5


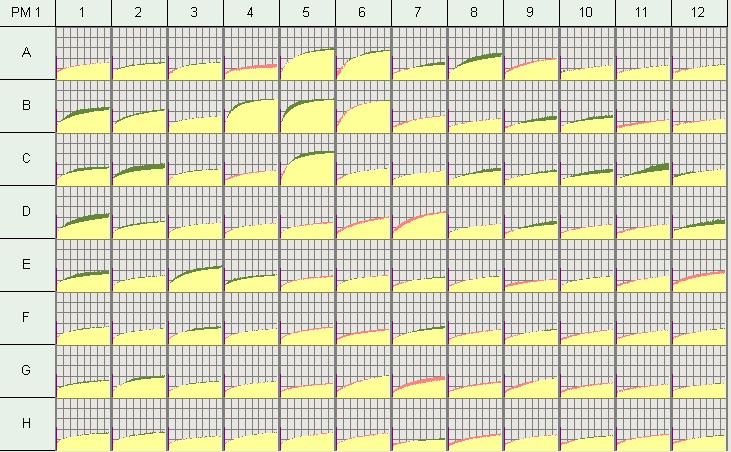


**Table.** Genes selected for this study with their aliases and functions. Information from the Gene database from NCBI website. (1)

| **Gene** | **Function** | **Association with syndromes/conditions** |
| --- | --- | --- |
| *ADSL*  Adenylosuccinate lyase (*ASL*, *AMPS*, *ASASE*) | Catalyzes two non-sequential reactions in the *de novo* purine biosynthetic pathway: the conversion of succinylaminoimidazole carboxamide ribotide (SAICAR) to aminoimidazole carboxamide ribotide (AICAR) and the conversion of adenylosuccinate (S-AMP) to adenosine monophosphate | Adenylosuccinase deficiency (ADSLD), a disorder marked with psychomotor retardation, epilepsy or autistic features (1) |
| *EP300*  E1A binding protein p300  (*p300*, *KAT3B*, *MKHK2*, *RSTS2*) | The adenovirus E1A-associated cellular p300 transcriptional co-activator protein functions as histone acetyltransferase regulating transcription via chromatin remodeling and is important in the processes of cell proliferation and differentiation. It mediates cAMP-gene regulation by binding specifically to phosphorylated CREB protein. This gene has also been identified as a co-activator of HIF1A (hypoxia-inducible factor 1 alpha), and thus plays a role in the stimulation of hypoxia-induced genes such as VEGF | Rubinstein-Taybi syndrome (typical facial features, microcephaly, broad thumbs and first toes, intellectual disability, and postnatal growth retardation) (1,2); may also play a role in epithelial cancer |
| *TNFRSF13C* TNF receptor superfamily member 13C (*BAFFR*, *CD268*, *CVID4*, *BAFF-R*, *BROMIX*, *prolixin*) | Encodes a type III transmembrane protein containing a single extracellular cysteine-rich domain and working as a receptor for B cell-activating factor (BAFF) playing a crucial role in BAFF-mediated mature B-cell survival | Schizophrenia, a disorder characterized by delusions, hallucinations, disorganized speech, grossly disorganized or catatonic behavior, and/or negative symptoms (*i.e.*, diminished emotional expression or avolition) (3) |
| *NAGA*  Alpha-N-acetylgalactosaminidase (*GALB*, *D22S674*) | Encodes the lysosomal enzyme alpha-N-acetylgalactosaminidase, which cleaves alpha-N-acetylgalactosaminyl moieties from glycoconjugates | Schindler disease types I (neurological problems, developmental regression, blindness, seizures and unresponsive as disease progresses) and II (type II also known as Kanzaki disease) (mild cognitive impairment, hearing loss, problems with peripheral nervous system and angiokeratomas) (4) |
| *A4GALT*  Alpha 1,4-galactosyltransferase (P blood group) (*P1*, *PK*, *Gb3S*, *P(k)*, *P1PK*, *A14GALT*, *A4GALT1*) | Catalyzes the transfer of galactose to lactosylceramide to form globotriaosylceramide, which has been identified as the P(k) antigen of the P blood group system. This protein, a type II membrane protein found in the Golgi, is also required for the synthesis of the bacterial verotoxins receptor | Caffey disease (bone disorder marked by excessive new bone formation) (5) |
| *TRMU*  tRNA 5-methylaminomethyl-2-thiouridylate methyltransferase (*MTO2*, *MTU1*, *TRMT*, *LCAL3*, *TRMT1*) | Encodes a mitochondrial tRNA-modifying enzyme which catalyzes the 2-thiolation of uridine on the wobble positions of tRNA(Lys), tRNA([37](#_ENREF_37)), and tRNA(Gln), resulting in the formation of 5-taurinomethyl-2-thiouridine moieties | Transient infantile liver failure (elevated liver enzymes, jaundice, vomiting, coagulopathy, hyperbilirubinemia and increased serum lactate) (1,6). Polymorphisms in this gene may also influence the severity of deafness caused by mitochondrial 12S ribosomal RNA mutations |
| *TUBGCP6* Tubulin gamma complex associated protein 6 (*GCP6*, *GCP-6*, *MCCRP*, *MCCRP1*, *MCPHCR*) | Encodes a large multi-subunit complex required for microtubule nucleation at the centrosome | Microcephaly and Chorioretinopathy, Autosomal Recessive, 1 and Autosomal Recessive Chorioretinopathy-Microcephaly Syndrome (Developmental disorder characterized by delayed psychomotor development and visual impairment, often accompanied by short stature) (7) |
| *SBF1*  SET binding factor 1 (*MTMR5*, *CMT4B3*, *DENND7A*) | Encodes a member of the protein-tyrosine phosphatase family. However, the encoded protein does not appear to be a catalytically active phosphatase because it lacks several amino acids in the catalytic pocket. This protein contains a Guanine nucleotide exchange factor (GEF) domain which is necessary for its role in growth and differentiation | Charcot-Marie-Tooth disease 4B3 (childhood onset of slowly progressing, demyelinating sensorimotor neuropathy, focally folded myelin sheaths in nerve biopsy, reduced nerve conduction velocities, distal muscle weakness and atrophy, and sensory loss) (8,9) |
| *ARSA* Arylsulfatase A (*ASA*, *MLD*) | Arylsulfatase A hydrolyzes cerebroside sulfate to cerebroside and sulfate | Metachromatic leukodystrophy (MLD), a progressive demyelination disease which results in a variety of neurological symptoms and ultimately death (1) |
| *SHANK3*  SH3 and multiple ankyrin repeat domains 3  (*PSAP2*, *SCZD15*, *PROSAP2*, *SPANK-2*, *DEL22q13.3*) | This gene is a member of the Shank gene family, encoding multidomain scaffold proteins of the postsynaptic density that connect neurotransmitter receptors, ion channels, and other membrane proteins to the actin cytoskeleton and G-protein-coupled signaling pathways. Shank proteins also play a role in synapse formation and dendritic spine maturation | Autism spectrum disorder (ASD, characterized by impairments in social interaction and communication, and restricted behavioral patterns and interests). Pathogenic variants have also been associated with Phelan-McDermid syndrome (PMS) and schizophrenia type 15. Some studies correlate it to seizures and dysmorphic features. (10,11) |

References:

1. Brown G.R., Hem V., Katz K.S., Ovetsky M., Wallin C., Ermolaeva O., Tolstoy I., Tatusova T., Pruitt K.D., Maglott D.R. *et al.* (2015) Gene: A gene-centered information resource at NCBI. *Nucleic Acids Res,* **43**, D36-D42.

2. Tang F., Li Z., Cheng X., Su N., Yan L., Gou P. and Gong C. (2019) Clinical and genetic analysis of two cases with Rubinstein-Taybi syndrome. *Zhonghua Yi Xue Yi Chuan Xue Za Zhi,* **36**, 886-889.

3. Tandon R., Gaebel W., Barch D.M., Bustillo J., Gur R.E., Heckers S., Malaspina D., Owen M.J., Schultz S., Tsuang M. *et al.* (2013) Definition and description of schizophrenia in the DSM-5. *Schizophr. Res.,* **150**, 3-10.

4. Kanekura T., Sakuraba H., Matsuzawa F., Aikawa S., Doi H., Hirabayashi Y., Yoshii N., Fukushige T. and Kanzaki T. (2005) Three dimensional structural studies of alpha-N-acetylgalactosaminidase (alpha-NAGA) in alpha-NAGA deficiency (Kanzaki disease): Different gene mutations cause peculiar structural changes in alpha-NAGAs resulting in different substrate specificities and clinical phenotypes. *J. Dermatol. Sci.,* **37**, 15-20.

5. Genetics Home Reference (2020) *Caffey Disease.*

6. Zeharia A., Shaag A., Pappo O., Mager-Heckel A.M., Saada A., Beinat M., Karicheva O., Mandel H., Ofek N., Segel R. *et al.* (2009) Acute infantile liver failure due to mutations in the TRMU gene. *Am. J. Hum. Genet.,* **85**, 401-407.

7. Martin C.A., Ahmad I., Klingseisen A., Hussain M.S., Bicknell L.S., Leitch A., Nurnberg G., Toliat M.R., Murray J.E., Hunt D. *et al.* (2014) Mutations in PLK4, encoding a master regulator of centriole biogenesis, cause microcephaly, growth failure and retinopathy. *Nat. Genet.,* **46**, 1283-1292.

8. Nakhro K., Park J.M., Hong Y.B., Park J.H., Nam S.H., Yoon B.R., Yoo J.H., Koo H., Jung S.C., Kim H.L. *et al.* (2013) SET binding factor 1 (SBF1) mutation causes Charcot-Marie-Tooth disease type 4B3. *Neurology,* **81**, 165-173.

9. Bohlega S., Alazami A.M., Cupler E., Al-Hindi H. and Ibrahim E. (2011) A novel syndromic form of sensory-motor polyneuropathy is linked to chromosome 22q13.31-q13.33. *Clin. Genet.,* **79**, 193-195.

10. Boccuto L., Lauri M., Sarasua S.M., Skinner C.D., Buccella D., Dwivedi A., Orteschi D., Collins J.S., Zollino M., Visconti P. *et al.* (2013) Prevalence of SHANK3 variants in patients with different subtypes of autism spectrum disorders. *Eur. J. Hum. Genet.,* **21**, 310-316.

11. Soorya L., Kolevzon A., Zweifach J., Lim T., Dobry Y., Schwartz L., Frank Y., Wang A.T., Cai G., Parkhomenko E. *et al.* (2013) Prospective investigation of autism and genotype-phenotype correlations in 22q13 deletion syndrome and SHANK3 deficiency. *Mol. Autism,* **4**, 18-18.
